# Epigallocatechin 3-Gallate Has a Neuroprotective Effect in Retinas of Rabbits with Ischemia/Reperfusion through the Activation of Nrf2/HO-1

**DOI:** 10.3390/ijms21103716

**Published:** 2020-05-25

**Authors:** Josué Rivera-Pérez, Martín Martínez-Rosas, César A. Conde-Castañón, Julia D. Toscano-Garibay, Nancy J. Ruiz-Pérez, Pedro L. Flores, Elvia Mera Jiménez, Javier Flores-Estrada

**Affiliations:** 1 Instituto de Neurobiología, Universidad Nacional Autónoma de Mexico (UNAM), Campus UNAM-Juriquilla, CP 76230 Querétaro, Mexico; biotecnologo1@hotmail.com; 2 Departamento de Fisiología, Instituto Nacional de Cardiología Ignacio Chávez, Juan Badiano 1, Sección 16, Tlalpan, CP 14080 Ciudad de Mexico, Mexico; martin5163@hotmail.com; 3 Departamento de Oftalmología, Centro Médico Nacional La Raza, Paseo de las Jacarandas S/N, La Raza, Azcapotzalco, CP 02990 Ciudad de Mexico, Mexico; dr.cconde@outlook.com; 4 División de Investigación, Hospital Juárez de Mexico, Av. Instituto Politécnico Nacional 5160, Magdalena de las Salinas, Gustavo A. Madero, CP 07760 Ciudad de Mexico, Mexico; julia.toscano@gmail.com (J.D.T.-G.); abril_cics@yahoo.com.mx (N.J.R.-P.); 5 Departamento de Instrumentación Electromecánica, Instituto Nacional de Cardiología Ignacio Chávez, Juan Badiano 1, Sección 16, Tlalpan, CP 14080 Ciudad de Mexico, Mexico; pelfoch07@yahoo.com.mx; 6 Laboratorio de Cultivo Celular, Escuela Superior de Medicina, Instituto Politécnico Nacional, Plan de San Luis y Díaz Mirón s/n, Casco de Santo Tomas, Miguel Hidalgo, CP 11340 Ciudad de Mexico, Mexico; elviamj@gmail.com

**Keywords:** retinal ischemia/reperfusion (rI/R), epigallocatechin-3-gallate (EGCG), high-mobility group Box 1 (HMGB1), nuclear factor erythroid 2-related factor 2 (Nrf2), heme oxygenase-1 (HO-1)

## Abstract

Retinal ischemia-reperfusion (rI/R) generates an oxidative condition causing the death of neuronal cells. Epigallocatechin 3-gallate (EGCG) has antioxidant and anti-inflammatory properties. Nonetheless, its correlation with the pathway of nuclear factor erythroid 2-related factor 2/heme oxygenase-1 (Nrf2/HO-1) for the protection of the retina is unknown. We aimed to evaluate the neuroprotective efficacy of single-doses of EGCG in rI/R and its association with *Nrf2/Ho-1* expression. In albino rabbits, rI/R was induced and single-doses of EGCG in saline (0–30 mg/kg) were intravenously administered to select an optimal EGCG concentration that protects from retina damage. To reach this goal, retinal structural changes, gliosis by glial fibrillary acidic protein (GFAP) immunostaining, and lipid peroxidation level by TBARS (thiobarbituric acid reactive substance) assay were determined. EGCG in a dose of 15 mg/kg (E15) presented the lowest levels of histological damage, gliosis, and oxidative stress in the studied groups. To determine the neuroprotective efficacy of E15 in a timeline (6, 24, and 48 h after rI/R), and its association with the Nrf2/HO-1 pathway, the following assays were done by immunofluorescence: apoptosis (TUNEL assay), necrosis (high-mobility group box-1; HMGB1), Nrf2, and HO-1. In addition, the *Ho-1* mRNA (qPCR) and lipid peroxidation levels were evaluated. E15 showed a protective effect during the first 6 h, compared to 24 and 48 h after rI/R, as revealed by a decrease in the levels of all damage markers. Nuclear translocation Nrf2 and HO-1 staining were increased, including *Ho-1* mRNA levels. In conclusion, a single dose of E15 decreases the death of neuronal cells induced by oxidative stress during the first 6 h after rI/R. This protective effect is associated with the nuclear translocation of Nrf2 and with an elevation of *Ho-1* expression.

## 1. Introduction

Retinal ischemia/reperfusion (rI/R) occurs in acute angle-closure glaucoma, retinal vascular occlusion, diabetic retinopathy, and retinopathy of prematurity, causing irreversible neuronal damage due to increased levels of reactive oxygen species (ROS). Oxidative stress activates microglia and causes the death of the ganglion cells by excitotoxicity of glutamate and by the production of nitric oxide [1,2]. Nonetheless, the retina has endogenous antioxidant mechanisms to maintain redox homeostasis, such as the nuclear factor erythroid 2-related factor 2/antioxidant responsive element (Nrf2/ARE) pathway [3]. Nrf2 is a transcriptional protein located in the cellular cytoplasm under physiological conditions, and that is degraded by the proteasome system. However, under an oxidative stimulus, Nrf2 is translocated to the nucleus where it interacts with small proteins, and binds to ARE to activate the transcription of antioxidant genes such as the nicotinamide adenine dinucleotide phosphate (NADPH) oxidase complex: quinone oxidoreductase 1, glutathione S-transferases (GSTs), gamma-glutamylcysteine ligase, and heme oxygenase 1 (HO-1) [3]. HO-1 displays immunomodulatory, anti-inflammatory, and cytoprotective properties, including the decrease of necrosis by reducing the high-mobility group box-1 (HMGB1) protein release [1,2,3,4,5,6]. In retinal damage models, activation of Nrf2 and HO-1 pathway (Nrf2/HO-1) show a neuroprotective effect, suggesting that it can be a meaningful therapeutic target [7,8]. Epigallocatechin 3-gallate (EGCG) is a flavonoid present in large amounts in the plant *Camellia sinensis* (green tea) and, due to its antioxidative and anti-inflammatory properties, it has been proposed as a protective agent in damaged retinas by several kinds of oxidative insults [9,10,11,12]. In cerebral ischemia models, EGCG increases Nrf2/HO-1 activity in a dose-dependent manner. However, high concentrations and long-term use could favor a pro-oxidant environment on retinas, rendering the determination of the optimal dose necessary [13].

On the other hand, the neuroprotective role of EGCG in association with the Nrf2/OH-1 regulation in the retina is still unknown. In the present study, we examined the protective effect of several single doses of intravenous EGCG in the early phase of rI/R. We also determined the efficacy of the optimal dose found to protect the retina from oxidative damage by I/R and sought its association with the levels of the endogenous antioxidants Nrf2 and HO-1. We hypothesized that EGCG would protect retinas from oxidative injury through the Nrf2/HO-1 modulation in a concentration and time-dependent manner.

## 2. Results

### 2.1. Effect of EGCG on Morphological Changes after Retinal Ischemia-Reperfusion (rI/R)

**Figure 1:**
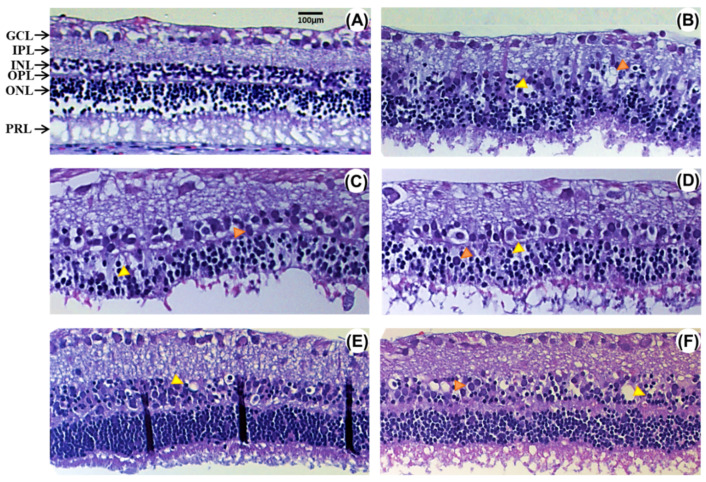
Effect of several concentrations of epigallocatechin 3-gallate (EGCG) on morphological changes induced by retinal ischemia/reperfusion (rI/R). (**A**) Sham group; (**B**) vehicle-treated rI/R (rI/R-veh); (**C**) treated with EGCG at 1.5 (rI/R-E1.5); (**D**) at 7.5 (rI/R-E7.5); (**E**) at 15 (rI/R-E15), and (**F**) at 30 mg/kg (rI/R-E30). Pinocytic nuclei and vacuolization are showed in yellow and orange arrowheads, respectively. Ganglion cell layer (GCL) shows vacuolated cells and nucleomegaly. In the outer plexiform layer (OPL), a vacuolated aspect with an eosinophilic area is observed. IPL, internal plexiform layer; INL, internal nuclear layer; outer nuclear layer ONL; and photoreceptor layer (PRL). Hematoxylin and eosin staining. Scale bar = 100 μm.

Figure 1 displays structural changes of retinas 48 h after ischemia-reperfusion (I/R) that were treated with vehicle (rI/R-veh) or with a single intravenous dose of EGCG at 1.5 (rI/R-E1.5), 7.5 (rI/R-E7.5), 15 (rI/R-E15), and 30 mg/kg (rI/R-E30). These changes are also shown in rI/R treated with the vehicle (Figure 1B), which are also compared to sham group (Figure 1A). It can be observed that the inner nuclear layer (INL) has cellular loss characterized by pinocytic nuclei, vacuolization, edema, and disorganization between the INL and the outer nuclear layer (ONL) in the vehicle treated group. There is also a decrease in ganglion cells (GCL), along with loss of cohesiveness with vacuolated aspect, nucleomegaly, and a thickening of the inner plexiform layer (IPL). The outer nuclear layer (ONL) was vacuolated with marked eosinophilia. In rI/R-E1.5 and rI/R-E7.5 groups (Figure 1C,D, respectively), the histological alterations were similar to the rI/R-veh group, except for a slightly better-conserved structure of the INL in rI/R-E7.5. The rI/R-E15 group showed marked preservation in the organization of GCL, IPL, and INL (Figure 1E). This effect was more evident than in the rI/R-E7.5 group. The rI/R-E30 group (Figure 1F) had a conserved neural layer structure, although it had more significant neuronal edema compared to rI/R-E15.

**Figure 2:**
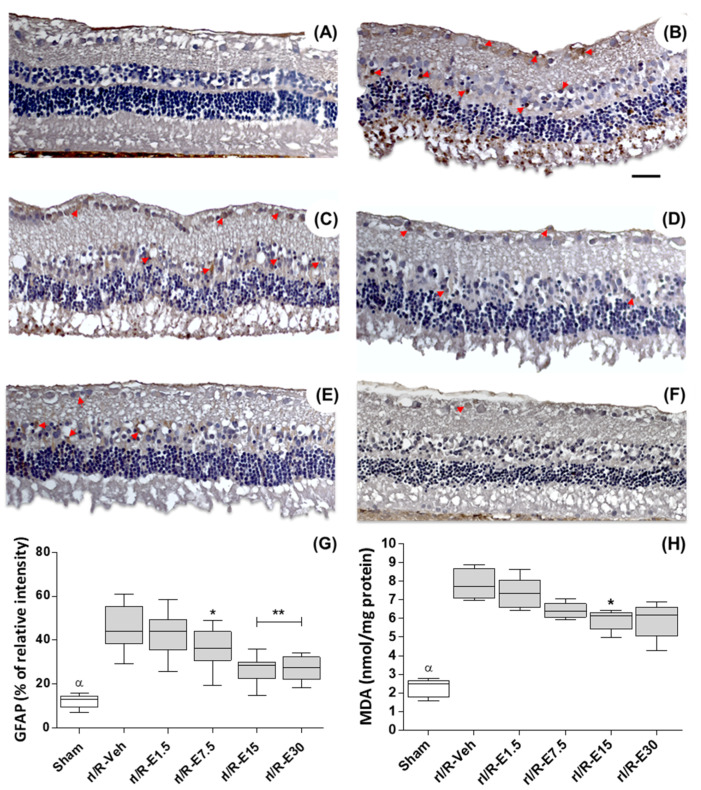
Effect of EGCG concentration on retinal protection at 48 h after rI/R. Gliosis retinal was measured by glial fibrillary acidic protein (GFAP) immunostaining (red arrows show representative cells). Lipid peroxidation levels were determined by malondialdehyde formation (MDA) assay. (**A**) sham group; (**B**) rI/R-veh; (**C**) rI/R-E1.5; (**D**) rI/R-E7.5; (**E**), rI/R-E15, and (**F**) rI/R-E30. The (**G**,**H**) show the Box-and-whisker plot (median, first-third quartile, minimum- maximum value) with the percentage of GFAP staining intensity and MDA formation levels, respectively. * *p* < 0.05, ** *p* < 0.01 compared to rI/R-veh, and ^α^ *p* < 0.01 compared to all groups. Scale bar = 100 μm.

The distribution and the staining intensity level of the glial fibrillary acidic protein (GFAP) were assessed to verify retinal damage at 48 h after rI/R. GFAP is a sensitive marker for retinal gliosis in response to neuronal degeneration. The rI/R-veh group shows evident gliosis manifested by an increased GFAP immunostaining in GCL, INL, and ONL (Figure 2B; red arrows) when compared with the sham group (Figure 2A). The Figure 2C to F show representative images of the distribution of GFAP staining treated by concentrations of EGCG (1.5, 7.5, 15, and 30 mg/kg, respectively). GFAP staining diminishes as the concentration of EGCG is increased in all retinal layers. There was a higher percentage relative intensity of GFAP staining in the rI/R-veh group (45.89 ± 9.88) when compared to the sham group (12.17 ± 2.87; *p* < 0.001) and there was not a significant alteration when compared to rI/R-E1.5 (42.15 ± 9.22). However, the GFAP staining percentages were significantly reduced in rI/R-E7.5 (36.08 ± 8.51; *p* < 0.05), in rI/R-E15 (26.91 ± 6.02; *p* < 0.01) and in rI/R-E30 (27.10 ± 5.39; *p* < 0.01). Figure 2G shows the plot (median, first-third quartile, minimum-maximum value) of GFAP staining percentage after rI/R in the groups studied.

Additionally, lipid peroxidation was quantified using the malondialdehyde (MDA) formation levels. MDA levels increased in rI/R-veh (7.86 ± 0.81) compared to the sham group (2.27 ± 0.49; *p* < 0.001). EGCG at 1.5 mg/kg (7.33 ± 0.85) did not show a difference with rI/R-veh (7.33 ± 0.85), whereas in EGCG treated groups ≥ 7.5 mg/kg, MDA levels were diminished: rI/R-E7.5 (6.42 ± 0.42; *p* < 0.05), rI/R-E15 (5.92 ± 0.57; *p* < 0.01), and rI/R-E30 (5.9 ± 0.98; *p* < 0.01). Figure 2H shows the plot (median, first-third quartile, minimum-maximum value) of the MDA formation levels after rI/R.

Because of the better preservation of the retinal structure after 48 h of rI/R and the lowest levels in GFAP and MDA in the groups treated with EGCG at 15 mg/kg, we decided its use as the optimal dose for the subsequent studies.

### 2.2. Neuroprotective Efficacy of EGCG at an Optimal Dose

For the evaluation of EGCG efficacy on the protection of rI/R, thirty-three right eyes treated with EGCG at 15 mg/kg (E15) (*n* = 15) and vehicle (*n* = 15) were divided into a timeline after rI/R, at 6, 24, and 48 h (*n* = 5 each). We also included a sham group at 48 h adding three eyes obtained from Section 4.2 (then, *n* = 6).

**Figure 3:**
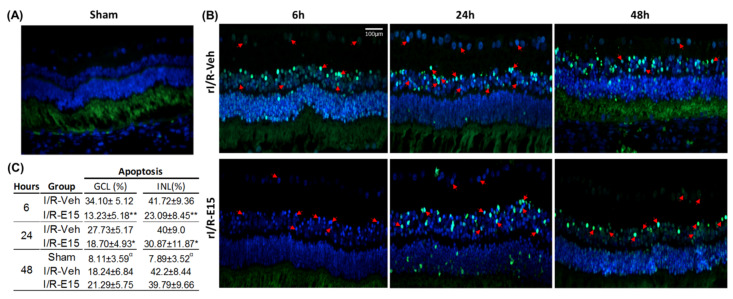
Neuroprotective effect of EGCG at 15 mg/kg (E15) in a timeline. Apoptosis was determined by terminal deoxynucleotidyl transferase dUTP nick end labeling (TUNEL) assay. (**A**) Sham group at 48 h after rI/R; (**B**) vehicle-treated groups at the 6, 24, and 48 h after rI/R (rI/R-veh; upper panel) and E15-treated group at the same times (rI/R-E15; bottom panel). Some apoptotic cells are highlighted with red arrows and their nuclei stained with DAPI (blue). In (**C**), the mean ± SD percentage of positive cells in the INL and GCL are shown. * *p* < 0.05 and ** *p* < 0.01 compared to rI/R-veh, and ^α^ *p* < 0.01 compared to rI/R-veh and rI/R-E15 groups, at 48 h. Scale bar = 100 μm.

To determine the neuroprotective effect of E15 in the timeline after rI/R, the terminal deoxynucleotidyl transferase dUTP nick end labeling (TUNEL) assay was performed as an apoptosis marker. At 6 h, in the rI/R-veh group, numerous cells in apoptosis were localized in GCL, INL, and ONL (Figure 3B, upper panel). At 6 h, in the rI/R-veh group, numerous cells in apoptosis were localized in GCL, INL, and ONL (Figure 3B, upper panel). In the E15-treated retinas, at 6 h after I/R, scarce apoptotic cells were found mainly in the INL and GCL (Figure 3B, bottom panel). Nevertheless, at 24 and 48 h, in the rI/R-veh and rI/R-E15 groups, apoptosis was found in all nuclear layers. Figure 3C shows a table with the apoptosis percentage in GCL and INL in the timeline. In the rI/R-E15 group, the apoptosis percentage was decreased at 6 and 24 h, compared to rI/R-veh (*p* < 0.01 and *p* < 0.05, respectively). However, 48 h after rI/R injury, the apoptosis percentage was similar between treated and untreated groups. The percentage of apoptotic cells in the E15-treated retinas was higher than in the sham group (*p* < 0.01). A sham group at 48 h was also included (Figure 3A).

**Figure 4:**
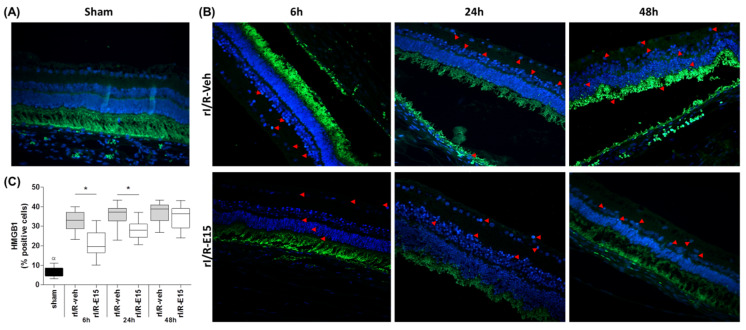
The protective effect from rI/R by E15 in the timeline. Necrosis was determined by high-mobility group box-1 (HMGB1) immunostaining assay. (**A**) Sham group at 48 h; (**B**) rI/R-veh at 6, 24, and 48 h after rI/R (upper panel) and E15-treated groups at the same times; (bottom panel). (**C**) Box-and-whisker plot (median, first-third quartile, minimum- maximum value) with the percentage of positive cells. Representative necrotic cells are shown with red arrows and their nuclei stained with DAPI (blue). * *p* < 0.05 compared to rI/R-veh ^α^ *p* < 0.01 compared to rI/R-veh and rI/R-E15 groups, at 48 h. Scale bar = 100 μm.

The neuroprotective role of E15 also was determined by the grade of necrosis after rI/R in the timeline using the high-mobility group box-1 (HMGB1) immunostaining as a marker. In rI/R-veh and rI/R-E15 groups, the stain of HMGB1 in the GCL, INL, and photoreceptor layers were observed (Figure 4B, upper and bottom panels). However, rI/R-15 at 6 h showed the lowest number of stained cells. In rI/R-E15 (at 6 and 24 h), the HMGB1 staining percentage was significantly lower when compared to rI/R-veh (*p* < 0.05) (32.91 ± 4.77 vs. 21.43 ± 6.31 at 6 h; 36.19 ± 4.78 vs. 28.16 ± 4.30 at 24 h). However, at 48 h, the HMGB1 percentage was not-significantly modified between rI/R-veh and rI/R-E15 (37.25 ± 4.96 vs. 34.89 ± 5.87, respectively). Figure 4C shows a plot (median, first-third quartile, minimum-maximum value) with the grade of retinal necrosis according to the percentage of HMGB1-positive cells after rI/R. A sham group was also included (Figure 4A).

### 2.3. E15 Activates Nrf2/HO-1 Antioxidative Pathway in rI/R

To determine if the neuroprotective effect of E15 in rI/R is related to the antioxidant Nrf2/OH-1 pathway, the nuclear staining of the Nrf2, and *Ho-1* expression, were evaluated. 

**Figure 5:**
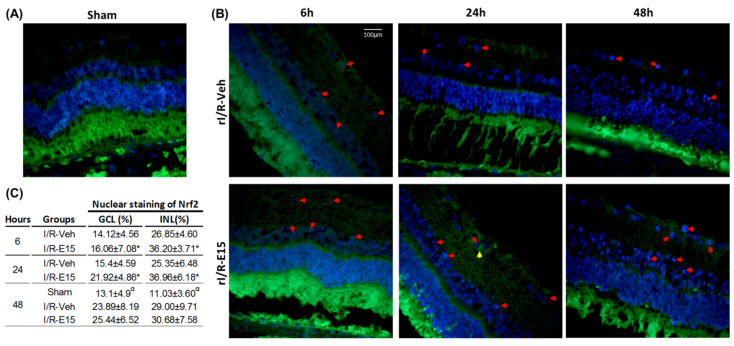
The antioxidant effect of E15 related to nuclear staining of nuclear factor E2-related factor 2 (Nrf2). The antioxidant effect was determined by nuclear immunostaining of Nrf2 after rI/R in the timeline. (**A**) Sham group at 48 h; (**B**) rI/R-veh at 6, 24, and 48 h after rI/R (upper panel), and rI/R-E15, at the same times (B; bottom panel). The nuclear (red arrows) and cytoplasmic (yellow arrows) Nrf2 staining and their nuclei stained with DAPI (blue) are showed. In (**C**), the table shows the nuclear percentage of NrF2 in INL and GCL (mean ± SD). * *p* < 0.05 compared to rI/R-veh and ^α^ *p* < 0.01 compared to rI/R-veh and rI/R-E15 groups, at 48 h. Scale bar = 100 μm.

The nuclear Nrf2 staining was found mainly in GCL and INL in the rI/R-veh and rI/R-E15 groups (Figure 5B; upper and bottom panels). Figure 5C shows a table with the nuclear Nrf2 percentage in GCL and INL in the timeline. In rI/R-E15, the nuclear Nrf2 percentage was higher at 6 and 24 h than the rI/R-veh (both *p* < 0.05). At 48 h after rI/R injury, the Nrf2 percentage showed a tendency to be higher when compared to rI/R-veh, but not significant difference was found. The nuclear Nrf2 percentage in the E15-treated retinas was higher than the sham group (*p* < 0.01). A sham group at 48 h was also included (Figure 5A).

**Figure 6:**
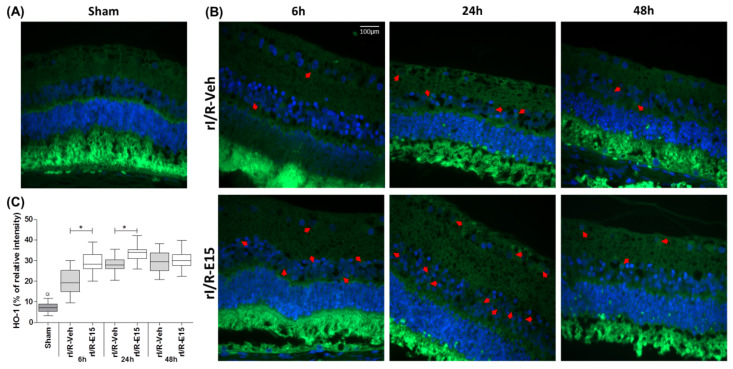
The antioxidant effect of E15 is related to heme oxygenase-1 (HO-1). The antioxidant effect was determined by immunostaining of HO-1 after rI/R in the timeline. (**A**) Sham group at 48 h; (**B**) rI/R-veh at 6, 24, and 48 h after rI/R (upper panel), and rI/R-E15 at the same times (bottom panel). Red arrows indicate its cytoplasmic distribution and their nuclei stained with DAPI (blue). In (**C**), shows a Box-and-whisker plot of (median, first-third quartile, minimum- maximum value) with the percentage of HO-1 staining intensity in all groups. * *p* < 0.05 compared to rI/R-veh and ^α^ *p* < 0.01 compared to rI/R-veh and rI/R-E15 at 48 h. Scale bar = 100 μm.

HO-1 immunofluorescence was performed to test if the changes in Nrf2 affected the consecutive effectors in the antioxidative pathway. In general, the HO-1 staining intensity in rI/R-veh and rI/R-E15 groups was limited to the perinuclear region of retinal cells (Figure 6B, upper and bottom panels). In rI/R-E15, at 6 and 24 h the staining intensity percentage was increased when compared with rI/R-veh (29.33 ± 4.72 and 19.84 ± 6.03; *p* < 0.05) and (33.50 ± 3.42 and 28.42 ± 2.90; *p* < 0.05) respectively. At 48 h after rI/R injury, rI/R-E15 did not show a significant difference with rI/R-veh (30.53 ± 4.00 and 29.39 ± 5.15, respectively). Figure 6C shows a plot (median, first-third quartile, minimum- maximum value) with the percentage of HO-1 staining intensity in rI/R-veh and rI/R-E15 in the timeline. A sham group at 48 h was also included (Figure 6A).

**Figure 7:**
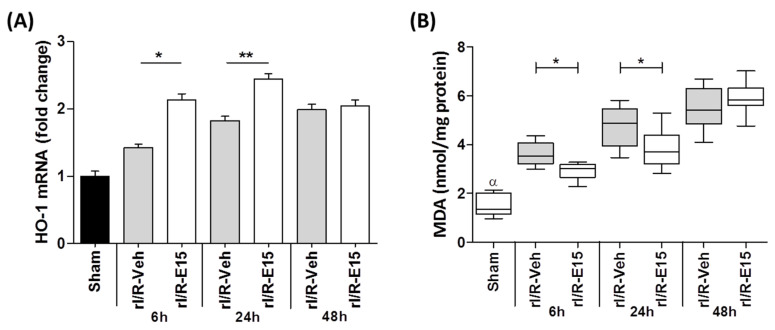
The E15 effect on *Ho-1* expression and lipid peroxidation level after rI/R in the timeline (6, 24, and 48 h). (**A**) Shows the fold changes of *Ho-1* mRNA quantified by qRT-PCR (mean ± SD) and (**B**) shows a Box-and-whisker plot (median, first-third quartile, minimum- maximum value) with MDA levels formation by TBARS assay. * *p* < 0.05, ** *p* < 0.01 compared to rI/R-veh. ^α^ *p* < 0.01 compared to rI/R-veh and rI/R-E15 groups, at 48 h.

The staining intensity of HO-1 in the retinas was further confirmed by quantifying its expression levels through qPCR (Figure 7A). Compared to rI/R-veh, the levels of *Ho-1* mRNA in rI/R-E15 at 6 and 24 h were increased (1.5 ± 13.31 and 1.5 ± 13.3 times, respectively) suggesting that E15-treated rI/R can activate the antioxidant pathway of Nrf2/HO-1. However, *Ho-1* mRNA levels between rI/R-veh and rI/R-E15 were not significantly different at 48 h.

### 2.4. Retinal Lipid Peroxidation in the Timeline

Nrf2 staining intensity and *Ho-1* expression levels were related to oxidative stress, which can be assessed by the formation of MDA at the 6, 24, and 48 h after rI/R and E15 effect. 

Figure 7B shows the plot the median, first-third quartile, minimum-maximum value of the MDA formation levels after rI/R in the timeline. A decrease of MDA levels in rI/R-E15 at 6 h (2.93 ± 0.34; *p* < 0.01) and 24 h (3.87 ± 0.74; *p* < 0.01) was observed when compared to rI/R-veh (3.60 ± 0.43 and 4.78 ± 0.77, respectively). The MDA level at 48 h after E15, did not show a statistically significant difference with rI/R-veh. MDA levels in the sham group at 48 h after rI/R were the lowest (1.50 ± 0.46) when compared to rI/R-E15 and rI/R-veh at that same time.

## 3. Discussion

The increase in reactive oxygen species and the death of retinal neurons impact retinal structure during ischemia/reperfusion (I/R) and threaten the loss of vision in humans [1,2].

Several animal models have been developed to study the retinal damage by stimuli as chemicals agents (e.g., N-methyl-D-aspartate), autoimmune response, mechanical stress, and transient retinal ischemia [14]. The partial optic nerve crush controlled by forceps is a model of mechanical stress-induced I/R, which has been used commonly in studies at long term (days–weeks) [15]. The elevated intraocular pressure (IOP) is also a model of I/R to study degeneration that occurs from the first minutes to 14 days after the injury.

Elevated IOP in rabbits causes an exudative retinal detachment characterized by a significant loss of ganglion cells, loss of the inner and outer nuclear layers, and photoreceptors degeneration like happens in humans [16,17,18,19]. Furthermore, the rabbit is a model suitable for surgery and provides enough tissue to carry out studies related to early retinal damage and to design therapeutic strategies. 

The diminishing in the production of reactive oxygen species (ROS) is a sine qua non condition to avoid I/R damage. Recent research has focused on studying flavonoids as antioxidant agents. In this regard, epigallocatechin 3-gallate (EGCG) and resveratrol show neuroprotective effects against cerebral injury by I/R [20] activating to Nrf2 and HO-1, which ameliorate the oxidative damage and decrease neuronal death.

Therefore, the present study investigated if EGCG protects from the retinal ischemic damage in a rabbit model and if this effect is related to the activity of the nuclear factor E2-related factor 2 (Nrf2) and heme oxygenase-1 (HO-1) pathway.

Previous studies showed in rI/R-induced animal models that EGCG has a neuroprotector role in the retina through its ROS-scavenger activity, inhibiting the glutamate-induced excitotoxicity and suppressing inflammatory factors release [9,10,11,12,21]. In models of cerebral ischemia and neuronal cultures, EGCG promotes neuronal protection regulated by the Nrf2/HO-1 pathway by attenuating both ROS production and nuclear factor κB (NF-κB) activation [22], which consequently suppresses the expression of TNFα, IL-1β, IL-6, and iNOS [23].

In this study, several intravenous concentrations at 48 h after rI/R were used to test the protective effect of EGCG. The rI/R increased the staining intensity of glial fibrillary acidic protein (GFAP) and lipid peroxidation levels. Moreover, there was an alteration in retinal layers structures of the vehicle-treated group and EGCG single-dose groups at minimal doses (1.5 and 7.5 mg/kg). The protective effects of EGCG were observed at 15 and 30 mg/kg compared to minimal doses. A single dose of 15 mg/kg (E15) was enough to improve the retinal alterations diminishing the retinal gliosis and oxidative stress. However, at 30 mg/kg, there were slight structural changes because of cell vacuolization in the inner nuclear layer and a moderated increase in GFAP staining and lipid peroxidation levels, suggesting that the neuroprotective role of EGCG follows a dose-limiting pattern. Ramachandran et al. [24] and Chu et al. [13] have described that the beneficial effect of EGCG appears to be a function of the dose, the administration route, and the duration of treatment. Higher EGCG or repeated doses in the long term might have a pro-oxidant effect [13,24]. In an ischemia model by middle cerebral artery occlusion in rats, the intraperitoneal injection of EGCG 20 mg/kg, had a neuroprotective effect [25]. However, EGCG at 50 mg/kg in the same model resulted in 50% of animals developing intracerebral hemorrhage [26]. 

On the other hand, nuclear translocation of Nrf2 and *Ho-1* expression was studied because both have been shown to have a protective role in the damage of retinal ganglion cells by I/R (IOP elevated) in rodent models, by antioxidant and anti-inflammatory mechanisms [27,28]. The up-regulation of NADPH: quinone oxidoreductase 1 and HO-1 by stabilization of Nrf2, can protect the retinal pigment epithelial cells from oxidative stress-induced necrosis [5,6]. This evidence suggests that the pharmacological activation of Nrf2 can be used as a therapeutic strategy since Nrf2 activation by synthetic derivatives of triterpenoid molecules showed antiproliferative, differentiating, and anti-inflammatory properties [29,30,31,32].

Here, in the ischemia induced-damage condition, E15 showed an increase in translocation of Nrf2 to the nucleus and the expression of *Ho-1*. Further, the apoptosis and lipid peroxidation levels, at 6 and 24 h after rI/R were diminished both in ganglion cells and the internal nuclear layer (INL). However, 48 h after retinal I/R, there were no significant differences in both groups, which shows that EGCG can be neuroprotective in a time-limited manner. This behavior could be due to its pharmacokinetics since EGCG has an elimination half-life of about 8.0 h [33,34,35].

High-mobility group box 1 (HMGB1) was determined because it has an essential role in retinal damage induced by I/R, promoting an inflammatory response and ganglion cell death and photoreceptors degeneration [36]. In rI/R-E15, EGCG protects the retina from ischemia diminishing HMGB1 levels in the photoreceptors and INL compared to the vehicle-treated rI/R. In this regard, in in vitro and in vivo studies, EGCG induces the aggregation of HMGB1 binding to this protein, protecting the host from sepsis and endotoxemia [37]. It has also been shown that EGCG activates several anti-oxidative mitochondrial mechanisms against ischemic injury [32]. Likewise, EGCG decreases I/R-induced cell death by activating the PI3K/Akt signaling pathway in H9C2 cells [38]. EGCG also protects human umbilical vein endothelial cells from atmospheric particulate matter 2.5-induced injury, promoting the expression of Nrf2/HO-1 through the ERK1/2 and p38 MAPK pathways [39].

In support of the hypothesis that the activation of signaling pathways by EGCG depends on a range of doses and is time-dependent, Chen et al. [40] demonstrated in cell lines, that EGCG is an activator of ERK, JNK, and p38 in the concentration range of 25 µM to 1 mM. In the same study, authors showed that the activation of caspase-3 was a relatively late event (peaked at 16 h), whereas activation of MAPKs happened much earlier (peaked at 2 h) [40]. It is possible that, at low concentrations of EGCG, activation of MAPK leads to ARE-mediated gene expression, including phase II detoxifying enzymes [39]. In contrast, at higher concentrations of EGCG, sustained activation of MAPKs such as JNK could lead to apoptosis. Therefore, EGCG in rI/R could induce mechanisms that activate antioxidant gene expression during the early injury, however this is dependent on doses too. It is important to consider that the model of the rabbit has some limitations to extrapolate our results to a clinical scenario. The rabbit eye presents morphological differences (lack of macula and a different retinal vasculature) [41] and the described molecular mechanisms for rI/R-induced damage for the human eye, have been not entirely characterized for this model. However, the objective was to demonstrate the neuroprotective effect of EGCG in rI/R determined by the grade of degeneration and cell death in retinal layers. Therefore, this model is suitable for our purposes.

## 4. Materials and Methods 

### 4.1. Animals

Animal experiments were performed using New Zealand male rabbits (weight, 2.0–2.5 kg). Water and standard food were available to the animals ad libitum. The care and management of experimental animals were carried out following the National Institutes of Health “Guide for the Care and Use of Laboratory Animals” and the standards described by the Association for Research in Vision and Ophthalmology (ARVO), and the Official Mexican Standard NOM-062-ZOO-1999. The “Hospital Juárez de México” Ethics Committee approved the study (under the amendment of HJM 2493/14-B on 16 August 2019). Briefly, surgical procedures performed under general anesthesia induced by intravenous injection of a mixture of ketamine-xylazine (35:5 mg/kg). Anesthesia was sustained by administering a dose of tiletamine: zolazepam (Zoletil-100; Virbac, Méx) as needed. Corneal analgesia was achieved using one to two drops of 5 mg/mL tetracaine HCl (Ponti ofteno^®^, Laboratorios Sophia, Mex). To euthanize animals, a lethal overdose of sodium pentobarbital was administered.

### 4.2. Retinal Ischemia/Reperfusion (rI/R) Model 

Under a surgical microscope, the anterior chamber of the right eye was cannulated at 2 mm above cornea limb, using a 30G-gauge needle, connected to a bottle containing a liter of saline. Retinal ischemia was induced by lifting the bottle approximately 2 m above the level of the eye, raising the intraocular pressure to 100 mmHg (Schiötz tonometer; Rudolf Riester GmbH., Jungingen, Germany) for 60 min. The ischemia was confirmed by retina blanching under the funduscopic examination. Sham-treated eyes underwent similar procedures without elevation of the saline bottle. Thirty min before the termination of the ischemia period, five milliliters each epigallocatechin 3-gallate (EGCG) dose (sham, 1.5, 7.5, 15, and 30 mg/kg) was administered intravenously in the marginal vein from the right ear (*n* = 5 each). After the ischemia period, the reperfusion was reached by removing the needle from the limbus, and a drop of moxifloxacin hydrochloride ophthalmic solution 0.5% (Vigamox^®^; Alcon Laboratories, Inc., Fort Worth, TX, USA) was applied. EGCG was purchased from Sigma-Aldrich (St. Louis, CA, USA) and dissolved in saline solution (vehicle). Before intravenous administration, the EGCG solution was sterilized by filtration using a 0.22-micron pore and used immediately.

### 4.3. Histological Evaluation

Forty-eight hours after rI/R, right eyes of the groups intravenously treated with vehicle, EGCG (1.5, 7.5, 15, and 30 mg/kg) (hereafter -E1.5, -E7.5, -E15, and -E30), and the sham group were enucleated and fixed in neutral formalin. Then, the eyeballs were sectioned in a vertical plane adjacent to the optic nerve, and the central region was processed and embedded in paraffin. The morphological alterations of retinas stained with hematoxylin and eosin (H&E) were evaluated. Other sections (2 microns in thickness) were mounted on slides, dewaxed, and rehydrated up to the antigenic retrieval (ImmunoDNA Retriever with Citrate, BioSB, Santa Barbara, CA, USA) and the slides were loaded into Shandon^®^ Sequenza^®^ chamber (Thermo Fisher Scientific, Inc., Waltham, MA, USA). Tissues were labeled with a polymer-based immunodetection system (Mouse/Rabbit PolyVue Plus™ HRP/DAB detection system, Diagnostic BioSystems, Pleasanton, CA, USA), according to the manufacturer’s instructions. Monoclonal mouse anti-Glial fibrillary acidic protein (GFAP) antibody (1:1000; MAB360; Chemicon International, Inc., Temecula, CA, USA) was applied. Subsequently, the enhancers Polyvue Plus^®^ and horseradish peroxidase (HRP) were added and incubated with DAB plus/chromogenic substrate.

### 4.4. Lipid Peroxidation Assay

About 5 mg of wet retina were collected, washed with saline solution, and homogenized in 300 microliters of RIPA lysis buffer for two min. The thiobarbituric acid reactive substance (TBARS) assay was used, according to the manufacturer’s instructions (OXItek-TBARS assay kit, Enzo Life Sciences, Farmingdale, NY, USA) to quantify of malondialdehyde (MDA) production during acid hydrolysis of the lipid peroxides. Briefly, the mixture was incubated at 95 °C for 1 h and cooled on ice for 10 min. Then, samples were centrifuged at 3000 rpm at 4 °C for 15 min, and the supernatant was used to measure MDA concentration (nmol/mg of protein) at 532 nm (EON, BioTek Instruments, Inc., Winooski, VT, USA). The concentration of MDA was calculated according to the average absorbance of the MDA from a standard curve.

### 4.5. Efficacy of EGCG Single-Dose Treatment on rI/R

Another thirty-three right eyes with rI/R treated with EGCG at 15 mg/kg (rI/R-E15) (*n* = 15) and vehicle-treated (*n* = 15) were divided at 6, 24, and 48 h (*n* = 5 each). A sham group at 48 h (*n* = 6, including three eyes obtained from Section 4.2) was analyzed. The tests were performed as described below.

### 4.6. Terminal Deoxynucleotidyl Transferase (TdT)-Mediated dUTP Nick End Labeling (TUNEL) Assay 

The death of retinal cells was detected according to the manual described by TUNEL assay using the In Situ Cell Death Detection Kit, POD (Roche diagnostics, Basel, Switzerland). Briefly, sections were permeabilized with citrate buffer 1× (pH 6.0) in a pressure cooker for 4 min. Then, incubated in equilibrium buffer (Tris-HCl, 0.1 M pH 7.5 containing 3% BSA and 20% normal bovine serum) for 30 min at room temperature, and rinsed twice with 0.1 M phosphate-buffered saline (PBS), pH 7.4. Finally, samples were incubated in terminal deoxynucleotidyl transferase enzyme at 37 °C for 60 min in the dark and washed three times in PBS. Nuclei were counterstained with 4′,6-Diamidine-2′-phenylindole dihydrochloride (DAPI).

### 4.7. Immunofluorescence

The immunofluorescent staining for high-mobility group box 1 (HMGB1), for nuclear factor E2-related factor 2 (Nrf2), and heme oxygenase-1 (HO-1) (all at 1:200 dilutions. Santa Cruz Biotechnology, Inc., Dallas, TX, USA) were performed. The slides were incubated with each primary antibody independently overnight at 4 °C. Sections then were washed three times with PBS and incubated with goat anti-mouse IgG (FITC)-conjugated secondary antibody (1:200; Santa Cruz, Biotechnology, Inc.) at room temperature for 1 h. Afterward, sections were washed and mounted in medium containing DAPI.

The histological image captures (200× magnification) were performed using an Axio Imager.A2 microscope with an integrated camera (Axiocam ICc5, Carl Zeiss Microscopy GmbH, Jena, Germany). The percentage for TUNEL, nuclear Nrf2 positive cells, GFAP, and staining intensity for HO-1 were measured using the Image-Pro Plus version 6.0 software (Media Cybernetics, Inc., Rockville, MD, USA). The analysis was performed in 5–8 sections from each specimen (for a total of 25 to 40 observations per group), proximal to the optical disk due to the higher density of ganglion cells. GCL, INL, and ONL were counted by two masked observers to evaluate staining interpretations. A third observer was required in cases of disagreement.

### 4.8. Semi-Quantitative PCR of Ho-1 mRNA

Total RNA was extracted from retinal tissue using TRIzol^TM^LS reagent (Life Technologies Corp., Carlsbad, CA, USA). Reverse transcription from 1 microgram of DNase I-treated RNA (Roche Applied Science, Mannheim, DE, USA) was performed using SuperScript^®^ II Reverse Transcriptase system (Life Technologies Corp., Carlsbad, CA, USA). The cDNA was subjected to real-time semi-quantitative PCR (qPCR) using SYBR Green analysis with a step one Real-Time PCR System (Applied Biosystems, Foster City, CA, USA). Two sets of primers previously designed genes were used to measure the expression levels of *Gapdh* [42] and *Ho-1* [43]. The temperature cycling protocol was performed according to Igarashi et al. [44] Relative gene expression was calculated using the standard curve method. The *Ho-1/Gapdh* ratio in sham retinal tissue was defined as 1.0. For experimental groups, the average value of the *Ho-1/Gapdh* ratio was obtained from 3 measurements. Each sample was run in triplicate. A melting curve analysis was also performed to ascertain the specificity of the amplified product. The expression for each gene was normalized to *Gapdh*. Expression was quantified as a fold-change using the ∆∆*C*t method.

### 4.9. Statistical Analyses

We used GraphPad Prism software (La Jolla, CA, USA; version 5.0). To *n* > 30 measures, the Kolmogorov-Smirnov normality test for all groups and its variables were analyzed. Values are shown as the mean with standard deviation (mean ± SD). In the Figure 2G,H, Figure 4C, Figure 6C and Figure 7A), the values are shown in box-and-whisker plots (Median, first-third quartile, minimum-maximum value). In the GFAP staining percentage, a one-way ANOVA test followed by Tukey’s test was performed. MDA formation levels were analyzed by the Kruskal-Wallis test, followed by Dunn’s test. To determine the effect of E15 in rI/R, in the timeline, data obtained from the rI/R-treated EGCG were compared with rI/R-veh and sham groups, and Bonferroni’s multiple comparison test was performed. *p* < 0.05 was considered statistically significant.

## 5. Conclusions

Our results show that the administration of a single dose of EGCG has a neuroprotective effect in an rI/R rabbit model by the activation of Nrf2/HO-1. The efficacy of E15 is better in the initial stages of damage (6–24 h) with an increase in nuclear translocation of Nrf2 and the *Ho-1* expression, suggesting this pathway is part of the protective antioxidant mechanisms associated with the significant decrease in retinal degeneration. We also suggest studying further the molecular mechanism (s) of its effects via the Nrf2/HO-1 pathway and its scavenger activity.

Finally, the useful dose of EGCG remains as a challenge for potential therapy development. For the long-term therapeutic effects, we suggest the necessity of determining the concentration and multiple dosage scheme of EGCG that should be administered to maintain the redox balance during the critical phase of retinal oxidative damage.

## Abbreviations

rI/R	Retinal ischemia/reperfusion
EGCG	Epigallocatechin-3-gallate
HMGB1	High-mobility group Box 1
Nrf2	Nuclear factor erythroid 2-related factor 2
HO-1	Heme oxygenase-1
GFAP	Glial fibrillary acidic protein
GCL	Ganglion cell layer
OPL	Outer plexiform layer
INL	Internal nuclear layer
IPL	Internal plexiform layer
ONL	Outer nuclear layer
PRL	Photoreceptor layer
